# Intravenous urography findings in women with ureteric fistula

**DOI:** 10.11604/pamj.2018.30.203.15345

**Published:** 2018-07-10

**Authors:** Ileogben Sunday-Adeoye, Kenneth Chinedu Ekwedigwe, Maradona Ehikioya Isikhuemen, Monday Osaze Eliboh, Emmanuel Nyan Yakubu, Babafemi Charles Daniyan

**Affiliations:** 1 National Obstetric Fistula Centre, Abakaliki, Ebonyi State, Nigeria; 2 Department of Obstetrics and Gynaecology, University of Benin Teaching Hospital, Benin City, Edo State, Nigeria

**Keywords:** Ureteric fistula, IVU, Abakaliki

## Abstract

**Introduction:**

Ureteric fistula is one of the major morbidities that can arise from pelvic surgeries. It mainly results from gynaecological and obstetric procedures. Intravenous urography is an imaging modality for the upper urinary tract. Its features may be suggestive of ureteric fistula and it is of great value when medicolegal issues arise. It is however expensive and requires expertise. There are other useful and cheap methods for evaluating ureteric fistula including the use of dye test. There is need to determine if IVU (Intravenous urography) should be recommended for women with this disease. The aim of this study was to determine the features of intravenous urography among women with ureteric fistula and therefore determine its relevance in the management of such patients.

**Methods:**

This was a retrospective study conducted at the National Obstetric Fistula Centre, Abakaliki between January 2012 and March 2017. All patients with ureteric fistula during the study period who were assessed with intravenous urography before surgery were included in this study.

**Results:**

The mean age was 38 ± 16 years. Twelve (92.3%) were Christians. IVU showed hydroureters in 46.15% hydronephrosis in 53.85%, non-functioning kidney in 46.15% and ureteric stricture in 7.69%. IVU gave an insight into the side with ureteric fistula except in one who had normal result.

**Conclusion:**

Hydronephrosis, hydroureters and silent (non-functioning) kidneys are features of IVU in women with ureteric fistulas, however these features are not pathognomonic for the disease.

## Introduction

Ureteric fistula usually arise from ureteral injury. Ureteric injury is one of the most feared complication of pelvic surgeries [1]. The main causes are gynaecological and obstetric procedures [2]. Ureteric injuries are often times iatrogenic, hence a source of medicolegal litigations [3]. The main method of presentation is urine leakage through the vagina. In the assessment of patients with ureteric fistula, IVU may be useful and can demonstrate the affected ureter. Intravenous urography helps in the assessment of the urinary tract especially its upper part. Features of ureteric fistula that can be seen on intravenous urogrphy are hydronephrosis, hydroureters and silent kidneys [4,5]. However, IVU is quite expensive. Another useful procedure for patients with ureteric fistula is vaginal speculum examination and dye test. A negative dye test with the presence of clear urine in the vagina is usually adjudged to be ureteric fistula [6]. It is cheap and does not require the attention of a specialist. There is therefore need to determine if patient assessment using IVU despite its cost should be prescribed for all patients with suspected ureteric fistula. The aim of management is to prevent damage of the affected part of the urinary tract by establishing the integrity of the urinary tract and restoration of normal continence. 3 Ureteroneocystostomy is the major modality for treatment. The aim of this study was to determine the features of intravenous urography among women with ureteric fistula with a view to determine its relevance in the management of such patients.

## Methods

This was a retrospective study conducted at the National Obstetric Fistula Centre, Abakaliki, South-East Nigeria between January 2012 and March 2017. The Fistula Centre is primarily for the management of genital fistula. It is also involved in other gynaecological procedures. It is located in South-East Nigeria. All patients with ureteric fistula who were evaluated with intravenous urography were included in the study. The study was approved by the Research and Ethics committee of the National Obstetric Fistula Centre, Abakaliki. Data was analysed using SPSS version 21.

## Results

**Figure 1:**
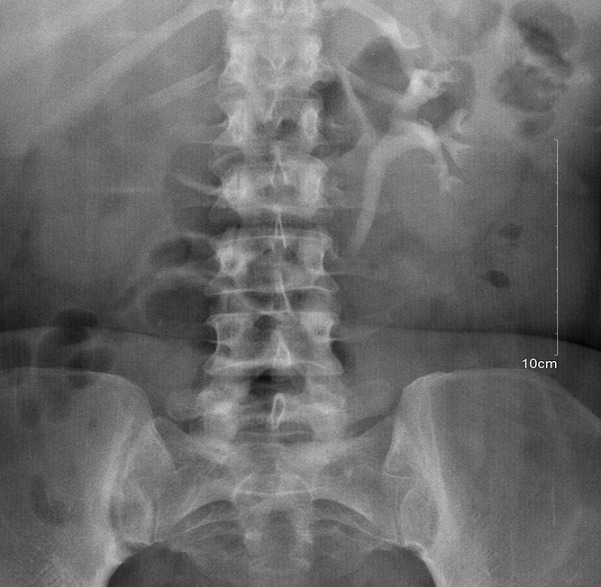
A patient with right hydronephrosis and right hydroureter

The mean age was 38 ± 16 years. Ten (76.9%) had secondary level of education and below (Table 1). Their parity ranged from 4 to 11 (mean parity was 4 ± 3). A total of 10 (76.9%) had emergency caesarean section (Table 2). The duration of symptoms ranged from one month to 14 years among women in the study population. IVU results indicated hydroureters in 6(46.15%) as shown in Figure 1, hydronephrosis in 7 (53.85%), non-functioning kidney in 6(46.15%), ureteric stricture in 1(7.69%) as shown in Table 3. IVU gave an insight into the side with ureteric fistula except in one who had normal result. They all had ureteric reimplantation and were followed up for three months without any complications.

**Table 1:** Sociodemographic variables

Variable	Frequency (%)
**Age**	
10 – 19	1(7.69)
20 – 29	2(15.38)
30 – 39	4(30.77)
40 – 49	5(38.46)
**50 and above**	1(7.69)
**Tribe**	
Igbo	9(69.37)
Ishan	1(7.69)
Yoruba	1(7.69)
Ibibio	1(7.69)
Urhobo	1(7.69)
**Religion**	
Christian	12(92.31)
Muslim	1(7.69)
**Occupation**	
Trading	8(61.54)
Civil servant	2(15.38)
Teaching	1(7.69)
Student	2(15.38)
**Level of Education**	
Primary	5(38.46)
Secondary	4(30.77)
Tertiary	3(23.08)
No Formal Education	1(7.69)
**Marital Status**	
Married	10(76.93)
Single	1(7.69)
Widow	1(7.69)
Divorced	1(7.69)

**Table 2:** Aetiological factor

	Frequency (%)
Emergency Caesarean section	10(76.93)
Congenital	1(7.69)
Vaginal hysterectomy	2(15.38)

**Table 3:** IVU findings

	Frequency (%)
Hydroureter	6(46.15)
Hydronephrosis	7(53.85)
Stricture in the ureter	1(7.69)
Non-functioning kidney / non visualization of the kidneys	6(46.15)
Normal findings	1(7.69)

## Discussion

This study was conducted to determine the role of IVU in patients with ureteric fistula. Bearing in mind the cost of IVU and the financial status of women with urogenital fistula, it has become necessary to investigate patient management without such investigation. Gynaecological and obstetric procedures remain the main causes of ureterovaginal fistula [2]. As shown in this study, ureteric fistulas mostly followed emergency caesarean sections and abdominal hysterectomies. Only one was congenital. In most cases, these patients usually go into labour at home or in the homes of traditional birth attendants. They subsequently arrive late to facilities that can offer caesarean section which makes it an emergency with its attendant risk of surgical errors. The features on IVU among women with ureteric fistula in this study were hydronephosis, hydroureter and non-functioning kidneys. Hydronephrosis and hydroureters are usually evidence of urinary stasis. The non visualization of contrast/non functioning kidney recorded in some patients may be explained by the fact there is distal obstruction in such patients and dye is not excreted. This result has to be interpreted with caution because it does not necessarily mean that the kidneys are in failure or truly non-functional. This may be a source of worry to the inexperienced medical practitioner. However in our practice, all such patients did well following ureteric reimplantation with no history suggestive of renal compromise. In a related study done among 30 women with ureterovaginal fistula, there were dilatation of the ureters, pelvis and calyces during intravenous pyelography in 22 patients [5]. In that study, the kidneys were not functioning in 3 patients while 5 had normal results. The authors of that paper reported nephrectomy in some cases. This was not the case in this article as no patient had nephrectomy including those with non-functioning kidneys. The patients presented in the index study did well following ureteric reimplantation. A vaginal speculum examination and negative dye test in the presence of clear urine in the vagina is usually adjudged to be ureteric fistula [6]. Obviously, this will usually not give an insight into the site of injury. Intravenous urography as seen in this study will most time give a clue as to the side of injury. In this study, the affected ureter suggested by the IVU was confirmed at surgery. In our setting where ureteroneocystostomy is routinely performed as an abdominal procedure, the relevance of knowing the side of the ureter affected is questionable since the pathology will be clearly visible when the bladder is opened as an initial step during the surgery. At least vaginal speculum examination and dye test alone can be used to diagnose a possible ureteric fistula. It is cheap and does not require expertise compared to IVU. Despite the usefulness of IVU in demonstrating ureteric pathologies, it may appear normal in the presence of a ureteric disease as seen in one of the patients. In another related study the authors also recorded some cases of ureteric fistula that had normal intravenous pyelography result [**5**]. However, this was not the case in another study where all 20 patients that had Ureteric pathologies were demonstrated by intravenous urography [7]. In this study, IVU were not done for patients after the ureters were reimplanted and we consider this to be a limitation. Also, a larger sample size is required to ultimately determine the role of IVU in the management of uretric fistulas.

## Conclusion

Obstetric and gynaecological injuries are the major causes of ureteric fistulas. Hydronephrosis, hydroureters and silent (non-functioning) kidneys are features of IVU in women with ureteric fistulas, however none of these features are pathognomonic for the disease. Results of IVU may be a useful guide in the pre-operative and intraoperative assessment of patients with ureteric fistula.

### What is known about this topic

- Intravenous urography is a method of investigation the upper urinary tract.

### What this study adds

- Role of intravenous urography in women with ureteric fistula;
- Intravenous urography do not appear to have pathognomonic features for ureteric fistula.

## Competing interests

The authors declare no competing interests.

## References

[1] Chalya PL, Massinde AN, Kihunrwa A, Simbila S (2015). Iatrogenic ureteric injuries following abdominopelvic operations: a 10-year tertiary care hospital experience in Tanzania. WJES.

[2] Tazi K, Moudouni S, Koutani A, Ibn Attya A, Hachimi M, Lakrissa A (2000). Uretero-vaginal fistula: therapeutic alternatives concerning 10 cases. Prog Urol.

[3] Farouk K, Najeeb R, Mohibullah, Mohsin B, Riaz H (2014). Ureteroneocystostomy versus ureteric stenting for the management of ureterovaginal fistula. JRMC.

[4] Benchekroun A, Lachkar A, Soumana A, Farih MH, Belahnech Z, Marzouk M, Faik M (1998). Ureterovaginal fistulas: 45 cases. Ann Urol(Paris).

[5] El Ouakdi M, Jlif H, Boujnah B, Ayed M, Zmerli S (1989). Ureterovaginal Fistula: a propos of 30 cases. J Gynecol Obstet Biol Reprod(Paris).

[6] Ileogben Sunday-Adeoye, Ehikioya Isikhuemen, Kenneth Ekwedigwe, Babafemi Daniyan (2017). Ureteric catheterization following ureteroneocystostomy in a low resource setting. Gynecology & Obstetrics.

[7] Al-Otaibi KM (2012). Ureterovaginal fistulas: the role of endescopy and a percutaneous approach. Urol Ann.

